# Postoperative recovery in peroral versus intravenous antibiotic treatment following laparoscopic appendectomy for complicated appendicitis: a substudy of a cluster randomized cluster crossover non-inferiority study

**DOI:** 10.1007/s00423-024-03491-w

**Published:** 2024-10-09

**Authors:** Ahmed Abdirahman Mohamud, Walid Zeyghami, Jakob Kleif, Ismail Gögenur

**Affiliations:** 1 Department of Surgery, Slagelse Hospital, Fælledvej 11, DK-4200 Slagelse, Denmark; 2 Department of Orthopedic Surgery, Copenhagen University Hospital Hvidovre, 2650 Hvidovre, Denmark; 3 Department of Surgery, Northzealand Hospital, 3400 Hilleroed, Denmark; 4 Center for Surgical Science, Zealand University Hospital, 4600 Koege, Denmark

**Keywords:** QoR-15, Quality of recovery, Recovery, Acute surgery, Laparoscopic appendectomy, Complicated appendicitis, Postoperative antibiotics

## Abstract

**Background:**

Acute appendicitis is the most common cause of abdominal pain requiring surgery, usually managed with laparoscopic appendectomy. In Denmark, the standard postoperative treatment for complicated cases involves intravenous antibiotics. This study compares peroral versus intravenous antibiotics in the context of fast-track surgery and Enhanced Recovery After Surgery (ERAS) protocols. Our objective is to evaluate the impact of peroral versus intravenous antibiotics on patient-reported outcomes following laparoscopic appendectomy for complicated appendicitis.

**Methods:**

This was a sub-study within a broader Danish cluster-randomized non-inferiority trial conducted at Zealand University Hospital, focusing on adult patients undergoing laparoscopic appendectomy for complicated appendicitis. Participants were randomized into two groups: one receiving a three-day course of peroral antibiotics and the other intravenous antibiotics after surgery. Recovery quality was assessed on the third postoperative day using the Quality of Recovery-15 (QoR-15) questionnaire.

**Results:**

The study included 54 patients, 23 in the peroral and 31 in the intravenous groups. The peroral group reported significantly better recovery outcomes, with higher QoR-15 scores (mean difference of 12 points, *p* < 0.001). They also experienced shorter hospital stays, averaging 47 h less than the intravenous group (*p* < 0.001). No significant differences between the groups were observed in readmissions or severe postoperative complications.

**Conclusions:**

Peroral antibiotic administration after laparoscopic appendectomy for complicated appendicitis significantly improves patient recovery and reduces hospital stay compared to intravenous antibiotics. These results advocate a potential shift towards peroral antibiotic use in postoperative care, aligning with ERAS principles.

**Trial Registration Number:**

ClinicalTrials.gov NCT04803422.

**Supplementary Information:**

The online version contains supplementary material available at 10.1007/s00423-024-03491-w.

## Background and aim

Acute appendicitis is a prevailing global surgical condition [1, 2]. In Denmark, the standard approach involves laparoscopic appendectomy, and nearly 40% of cases receive an intraoperative diagnosis of complicated appendicitis [3]. Following surgery for complicated appendicitis the national practice is a three-day regimen of postoperative intravenous antibiotics [3].

Peroral antibiotics may be non-inferior to intravenous antibiotics regarding postoperative intraabdominal abscess formation and wound infections. Such a shift in route of administration will very likely decrease postoperative admission time and health care expenses [3]. The impact on patient reported outcomes remain unknown. In line with the concepts of fast-track surgery and Enhanced Recovery After Surgery (ERAS) protocols, early mobilization and discharge may improve patient recovery in in this cohort of patients [4, 5].

The Quality of Recovery-15 (QoR-15) has been validated as a patient reported outcome measurement of postoperative recovery after various types of surgeries including laparoscopic appendectomy [6–11].

The effect of using a peroral route of administration versus intravenous administration of postoperative antibiotics after laparoscopic surgery for complicated appendicitis is currently being investigated in a nationwide cluster randomized non-inferiority trial in Denmark [12]. In the context of this ongoing trial, we aimed to evaluate patient-reported recovery using the QoR-15 in the two cohorts receiving either oral or intravenous antibiotic after laparoscopic appendectomy for complicated appendicitis.

## Methods

### Trial design and settings

This study constitutes a sub-study within an ongoing cluster-randomized, cluster-crossover non-inferiority pragmatic trial focusing on assessment of divergent standard postoperative treatments involving peroral and intravenous antibiotic therapies for complicated appendicitis following laparoscopic appendectomy and we explained the methods in detail in our protocol article [12]. Table 1 gives a detailed overview of the study phases and treatment protocols. Adherence to the principles outlined in the Helsinki Declaration [13] was followed, and necessary approvals were obtained from the Regional Ethics Committee of the Capital Region of Denmark, the Danish Data Protection Agency, as well as registration on ClinicalTrials.gov (NCT04803422).

**Table 1:** Detailed Study Phases and Treatment Protocols for QoR-15 study

Phase	Duration	Treatment Protocol	Hospital Stay	Antibiotic Regimen
Intravenous Antibiotics	6 months	IV antibiotics for 3 days	3 days	Metronidazole (500 mg) and Piperacillin/Tazobactam (4000 mg/500 mg) three times daily. In case of allergy to penicillin: Metronidazole (500 mg) and Cefuroxim (500 mg) three times daily
Peroral Antibiotics	6 months	Oral antibiotics for 3 days	Immediate discharge as soon as clinical discharge criteria were met	Amoxicillin/Clavulanic Acid (500 mg/125 mg) and Metronidazole (500 mg) three times daily. In case of allergy to penicillin: Ciprofloxacin (500 mg) two times daily and Metronidazole (500 mg) three times daily

Table 1 gives a detailed overview of study phases and treatment protocols for the QoR-15 study

We conducted the trial at the Department of Surgery within Zealand University Hospital in Denmark, a department attending to approximately 265,000 patients, including those with acute surgical conditions. Reporting adheres to the CONSORT statement for randomized clinical trials and its extensions about cluster trials, cross-over studies, and non-inferiority studies [14–16].

### Participants

We focused on patients undergoing laparoscopic appendectomy for complicated appendicitis characterized as gangrene, periappendicular abscess, perforation and peritonitis. We included individuals aged 18 years or older who were treated with either oral or intravenous antibiotics following laparoscopic appendectomy for complicated appendicitis. An additional inclusion criterion was the participation of patients in the QoR-15 survey, a measure of post-operative recovery administered through a telephone call on postoperative day three. Patients who were treated with oral antibiotics were discharged immediately after surgery as soon as clinical discharge criteria were met and patients treated with intravenous antibiotics were admitted for three days. All patients in both groups were contacted on postoperative day three in order to complete the QoR-15 survey.

We excluded patients who did not go through laparoscopic appendectomies, those with psychiatric conditions that could impede their participation in the study, and patients that could not understand the written or oral study information in Danish. Participants were required to provide oral and written informed consent before being included in the study. The data collected encompassed a range of variables, including sex, date of birth, the date and time of the start and conclusion of the surgery, ASA (American Society of Anesthesiologists) class, and the date and time of hospital admission and discharge. Additionally, the length of the hospital stay in hours, and complications of grade ≥ 3a according to the Clavien-Dindo Classification were recorded at 30 days postoperatively.

### Intervention

In the PIPA trial at Zealand University Hospital, standard laparoscopic appendectomy using a three-port approach was conducted. We structured the trial into two distinct six-month phases (table 1). The first phase of the trial entailed a three-day postoperative intravenous antibiotic regimen administered in an inpatient setting, succeeded by a second phase involving a three-day peroral antibiotic regimen managed in an outpatient setting. During the first six-month period, patients undergoing laparoscopic appendectomy received intravenous antibiotics and were hospitalized for three days. In the following six months, patients were discharged after they met clinical discharge criteria and were prescribed a three-day course of oral antibiotics. The oral antibiotic regimen commenced postoperatively once the patient could tolerate oral intake.

The pre- or intraoperative antibiotic protocol for both uncomplicated and complicated appendicitis included a single dose of intravenous Metronidazole (1000 mg) and Piperacillin/Tazobactam (4000 mg/500 mg). The regimen was altered for patients with penicillin allergies to a single dose of intravenous Metronidazole (1000 mg) and Cefuroxime (1500 mg).

In the postoperative period, the peroral regimen involved administering oral Amoxicillin/Clavulanic Acid (500 mg/125 mg three times daily) and Metronidazole (500 mg three times daily) for three days. For patients allergic to penicillin, the alternative regimen included Ciprofloxacin (500 mg twice daily) and Metronidazole (500 mg three times daily). The intravenous arm comprised Metronidazole (500 mg three times daily) and Piperacillin/Tazobactam (4000 mg/500 mg three times daily) for three days, with an alternative regimen of Metronidazole (500 mg three times daily) and Cefuroxime (1500 mg three times daily) for those with penicillin allergies.

### Outcomes

The primary outcome was evaluated through patient-reported postoperative recovery, assessed on the third postoperative day using the QoR-15 questionnaire. The QoR-15 questionnaire can be found in Supplementary Fig. 1. The QoR-15 questionnaire assesses recovery across five domains using 15 items, each rated on an 11-point scale (0–10). The total score ranges from 0 (indicating poor recovery) to 150 (indicating high recovery quality). The QoR-15 questionnaire has undergone validation, including its adaptation to the Danish language [6], and incorporates severity classifications: 0–89 (poor), 90–121 (fair), 122–135 (good), and 136–150 (excellent) [17]. Previous studies defined the minimal clinically important difference (MCID) of QoR-15 as 8 and recent update on MCID of the QoR-15 has changed the definition to 6 [18, 19]. In our analysis, we followed the updated definition of MCID to 6. Secondary outcome was time from end of surgery to discharge, readmissions within 30 days of discharge, and postoperative complication Clavien-Dindo grade 3 or above within the first 30 days postoperative.

### Randomization

In the PIPA trial [12], cluster randomization at a 1:1 ratio was executed using an electronically generated sequence via R software, with allocation not concealed post-randomization. The trial comprised two six-month phases of postoperative antibiotic treatment in patients undergoing laparoscopic appendectomy for complicated appendicitis. Participants were randomized to either a six-month intravenous antibiotic regimen followed by a six-month peroral regimen or a six-month peroral regimen followed by an intravenous one.

At Zealand University Hospital, the trial's first phase, involving intravenous antibiotic administration, spanned from 1 August 2022 to 31 January 2023, followed by the peroral antibiotic phase from 1 February 2023 to 31 July 2023.

### Sample size

Because of the explorative nature of this study and the fixed timeframe of the study we did not perform a sample size calculation. We expected to include more than 50 patients.

### Statistical methods

Continuous data are presented as median (interquartile range) or mean (standard deviation). Categorical data are expressed as counts (%). Fisher's exact test or Kruskal–Wallis was used as appropriate.

Both the primary and secondary outcomes were analyzed using linear regression in a univariable model, and as a sensitivity analysis in a multivariable model including sex, age, ASA group, and duration of surgery. Results are presented as mean with corresponding 95% confidence interval. Model assumptions were checked using residual diagnostics.

All available data was used. Imputation for missing data was not performed. A p-value equal or below 0.05 was considered significant. Statistical analyses were performed using R software (version 4.1.3) and RStudio software (version 1.4.1564).

## Results

**Fig. 1:**
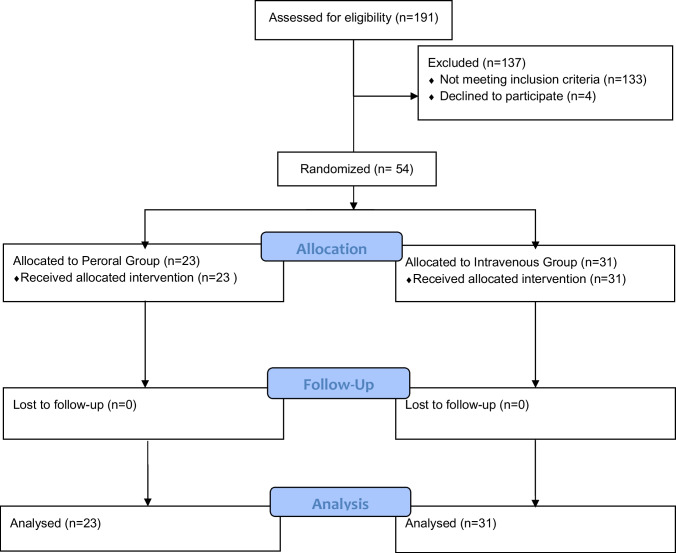
CONSORT Flow Diagram of the QoR-15 study

From August 1 2022 to July 2023, we included 54 patients who underwent laparoscopic appendectomy for complicated appendicitis and were allocated into two postoperative antibiotic treatment groups, with 23 patients in the peroral group and 31 patients in the intravenous group (Fig. 1). The baseline characteristics and intraoperative data are illustrated in Table 2.

**Table 2:** Baseline characteristics

Parameter	Intravenous Group (*n* = 31)	Peroral Group (*n* = 23)	*p*-value
Median Age (years)	65.00 (IQR: 41.50–70.50)	47.00 (IQR: 36.00–70.00)	0.234
Male Sex—No (%)	14 (45.2%)	15 (65.2%)	0.175
ASA Physical Status – No. (%)			0.030
1	8 (25.8%)	13 (56.5%)	
2	20 (64.5%)	7 (30.4%)	
3	3 (9.7%)	3 (13.0%)	
Median Time from Admission to Surgery (hours)	12.57 (IQR: 9.32–14.93)	11.95 (IQR: 9.27–19.65)	0.495
Median Duration of Surgery (hours)	0.97 (IQR: 0.82–1.17)	1.18 (IQR: 0.99–1.42)	0.041
Perforated appendicitis (including appendicitis with periappendicular abscess – No. (%)	30 (96,8)	18 (78,3)	0.073
Diffuse purulent peritonitis – No. (%)	13 (41,9)	4 (17,4)	0.077
Gangrenous appendicitis – No (%)	10 (32,3)	8 (34,8)	1.000

Table 2 displays the study participants' baseline demographic and clinical characteristics, categorized into the Intravenous Group (31 patients) and the Peroral Group (23 patients). It outlines key parameters such as median age, gender proportion, ASA physical status, time from admission to surgery, duration of surgery and cause of complicated appendicitis(perforated appendicitis with periappendicular abscess, diffuse purulent peritonitis and gangrenous appendicitis). Values are presented as medians with interquartile ranges (IQR) for continuous variables and as numbers with percentages for categorical variables. This table provides essential baseline data for comparative analyses between the two patient groups and *p*-values. *Abbreviations*: *IQR* Interquartile Range; *ASA* American Society of Anesthesiologists; *SMD* Standardized Mean Difference

### Quality of recovery

**Fig. 2:**
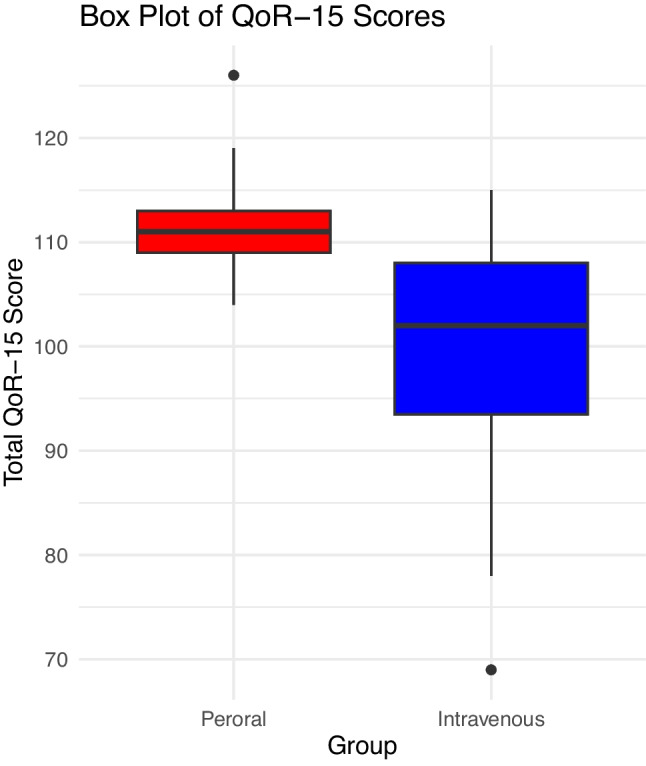
Boxplot of QoR-15, postoperative day 3

The QoR-15 scores in the two groups are visually presented in Fig. 2 showing a higher median score of 111 in the peroral group versus 102 the intravenous group.

Patients in the peroral group reported a better quality of recovery with a higher mean QoR-15 score of 111 (95% CI: 108;115) compared to patients in the intravenous group with a mean QoR-15 score 99 (95% CI: 95;105) resulting in a difference of 12 (95% CI: 7;17, *p* =  < 0.001) exceeding the MCID of 6. After controlling for sex, age, ASA score, and duration of surgery in a multivariable analysis, the peroral group still reported a higher mean QoR-15 score with a difference of 10 (95% CI: 5;16, *p* =  < 0.001) (Table 3).

**Table 3:** Multivariable analysis of QoR-15 scores on postoperative day 3

Characteristic	Mean Difference in points	95% CI	*p*-value
Intravenous antibiotics	Reference	—	—
Peroral antibiotics	10	5, 16	< 0.001
Female	Reference	—	—
Male	-1	-6, 4	0.7
Age – years	0	0, 0	0.9
ASA 1	Reference	—	—
ASA 2	-3	-9, 4	0.4
ASA 3	0	-9, 9	> 0.9
Duration of surgery—hours	3	-1, 8	0.14

This table presents the results of a multivariable analysis assessing the impact of various factors on QoR-15 scores on the third-day post-surgery. The analysis includes groups based on administration route, gender, age, ASA physical status, and surgery duration. *Abbreviations*: *CI* Confidence Interval; *IQR* Interquartile Range; *ASA* American Society of Anesthesiologists

### Time to discharge

Univariable analysis showed that time to discharge for the peroral group were 37 h (95% CI: 24;49) compared to patients in the intravenous group of 84 h (95% CI:68;100) with a difference of 47 h (95% CI: 31;63, *p* =  < 0.001). Multivariable analysis showed that patients in the peroral group were discharged an average 52 h (95% CI: 35;68, *p* =  < 0.001) earlier than patients in the intravenous group (Table 4) after adjusting for sex, age, ASA group, and duration of surgery.

**Table 4:** Multivariable analysis of time to discharge between peroral and intravenous groups

Characteristic	Mean Difference in points	95% CI	*p*-value
Intravenous antibiotics	Reference	—	—
Peroral antibiotics	-52	-68, -35	< 0.001
Female	Reference	—	—
Male	-6.5	-23, 9.6	0.4
Age – years	-0.21	-0.66, 0.23	0.3
ASA 1	Reference	—	—
ASA 2	-15	-35, 4.6	0.13
ASA 3	18	-9.6, 47	0.2
Duration of surgery—hours	-8.8	-23, 5.1	0.2

This table illustrates the multivariable analysis of time to discharge comparing the Peroral and Intravenous groups. The analysis evaluates the impact of various factors, including group type, gender, age, ASA physical status, and surgery duration on discharge timing. *Abbreviations*: *CI* Confidence Interval; *IQR* Interquartile Range; *ASA* American Society of Anesthesiologists

### Readmission

The route of antibiotic treatment was not associated with readmission (OR: 0.51, 95% CI: 0.09;2.56, *p* = 0.4). When controlling for sex, age, ASA score, and duration of surgery, the route of antibiotic treatment was still not associated with readmission (OR: 0.32, 95% CI: 0.05;1.83, *p* = 0.2).

### Postoperative complications Clavien-Dindo grade ≥ 3

Our analysis revealed no significant association between the route of administration between peroral versus intravenous and the incidence of postoperative complications as classified by Clavien-Dindo grade 3 or higher. In the univariable model, the OR for postoperative complications associated with intravenous administration, compared to peroral, was 1.52 (95% CI: 0.14;33.9, *p* = 0.7). After adjusting for sex, age, ASA score, and duration of surgery, the association remained statistically non-significant with an OR of 1.47 (95% CI: 0.08;46.5, *p* = 0.8).

## Discussion

We present an exploratory analysis within a more extensive cluster-randomized non-inferiority trial [12], examining the impact of peroral versus intravenous antibiotics on postoperative recovery following laparoscopic appendectomy for complicated appendicitis. Our results revealed a significant difference in the QoR-15 scores, favoring the peroral antibiotic group.

Our study observed a significant difference in the quality of recovery between the peroral and intravenous administration groups. Patients in the peroral group demonstrated a higher QoR-15 score compared to the intravenous group. The observed difference in the mean QoR-15 score was 12 points and the lower bound of the 95% confidence interval was above, which the established MCID of 6. This finding indicates a statistically and clinically significant better recovery in patients receiving peroral administration than those receiving intravenous treatment [18, 19]. The results are consistent with ERAS protocols, which advocate for less invasive management and encourage early mobilization for better patient outcomes [4, 5].

Patients receiving peroral antibiotics showed shorter hospital stays, indicating a more effective use of hospital resources. This is significant considering current efforts to improve hospital efficiency and reduce costs. Our study underscores the value of peroral antibiotics in potentially decreasing hospitalization time, aligning with healthcare goals of resource optimization.

The study's results may have significant implications for clinical practice, advocating for a paradigm shift towards peroral antibiotic administration after laparoscopic appendectomy for complicated appendicitis. The increased quality of recovery and shorter hospital stay associated with peroral antibiotics may contribute to enhanced patient satisfaction and lower healthcare costs. Peroral antibiotics may be non-inferior compared to intravenous antibiotics with regards to preventing postoperative abscess formation [3].

While offering valuable insights, this study has several limitations. The exploratory nature of this analysis and the absence of a predetermined sample size calculation may limit the precision and reliability of the findings. The single-center setting of the study at Zealand University Hospital may only partially represent diverse patient populations. The short follow-up period restricts our ability to assess long-term outcomes and potential delayed complications associated with the two antibiotic administration routes.

However, our ongoing PIPA trial, a cluster-randomized non-inferiority trial, may shed further conclusive light on whether peroral antibiotics are non-inferior compared to intravenous antibiotics in regards to infectious complications after surgery for complicated appendicitis [12].

In conclusion, the study offers evidence supporting the use of peroral antibiotics over intravenous antibiotics for improving the quality of recovery in patients undergoing laparoscopic appendectomy for complicated appendicitis.

## Supplementary Information

Below is the link to the electronic supplementary material.

Supplementary file1 (DOC 35 KB)

## Data Availability

No datasets were generated or analysed during the current study.

## Declarations

### Whether the paper is based on a previous communication to a society or meeting (with full details)

No.
