# Supplementary material for: Postoperative recovery in peroral versus intravenous antibiotic treatment following laparoscopic appendectomy for complicated appendicitis: a substudy of a cluster randomized cluster crossover non-inferiority study
Source: Langenbecks Arch Surg. 2024 Oct 9;409(1):303. doi: 10.1007/s00423-024-03491-w (PMC11461574; doi:10.1007/s00423-024-03491-w)
Supplement: Supplementary file 1 — Supplementary file1 (DOC 35 KB) [file 423_2024_3491_MOESM1_ESM.doc]

**Supplementary figure 1. The QoR-15 questionnaire.**

**PART A**

***How have you been feeling in the last 24 hours?***

(0 to 10, where: 0 = none of the time [poor] and 10 = all of the time [excellent])

1. Able to breathe easily None of All of

the time 0 1 2 3 4 5 6 7 8 9 10 the time

1. Been able to enjoy food None of All of

the time 0 1 2 3 4 5 6 7 8 9 10 the time

1. Feeling rested None of All of

the time 0 1 2 3 4 5 6 7 8 9 10 the time

1. Have had a good sleep None of All of

the time 0 1 2 3 4 5 6 7 8 9 10 the time

1. Able to look after personal None of All of

toilet and hygiene unaided the time 0 1 2 3 4 5 6 7 8 9 10 the time

1. Able to communicate with None of All of

family or friends the time 0 1 2 3 4 5 6 7 8 9 10 the time

1. Getting support from hospital None of All of

doctors and nurses the time 0 1 2 3 4 5 6 7 8 9 10 the time

1. Able to return to work or None of All of

usual home activities the time 0 1 2 3 4 5 6 7 8 9 10 the time

1. Feeling comfortable and in None of All of

control the time 0 1 2 3 4 5 6 7 8 9 10 the time

1. Having a feeling of general None of All of

well-being the time 0 1 2 3 4 5 6 7 8 9 10 the time

**PART B**

***Have you had any of the following in the last 24 hours?***

(10 to 0, where: 10 = none of the time [excellent] and 0 = all of the time [poor])

1. Moderate pain None of All of

the time 10 9 8 7 6 5 4 3 2 1 0 the time

1. Severe pain None of All of

the time 10 9 8 7 6 5 4 3 2 1 0 the time

1. Nausea or vomiting None of All of

the time 10 9 8 7 6 5 4 3 2 1 0 the time

1. Feeling worried or anxious None of All of

the time 10 9 8 7 6 5 4 3 2 1 0 the time

1. Feeling sad or depressed None of All of

the time 10 9 8 7 6 5 4 3 2 1 0 the time
